# The Use of Practical Measures to Determine Body Composition of Older People

**DOI:** 10.21315/mjms2023.30.5.11

**Published:** 2023-10-30

**Authors:** Pakwipa Chokphukiao, Puttipong Poncumhak, Roongnapa Intaruk, Thiwabhorn Thaweewannakij, Charoonsak Somboonporn, Sugalya Amatachaya

**Affiliations:** 1 School of Physical Therapy, Faculty of Associated Medical Sciences, Khon Kaen University, Khon Kaen, Thailand; 2 Improvement of Physical Performance and Quality of Life (IPQ) Research Group, Khon Kaen University, Khon Kaen, Thailand; 3 Department of Physical Therapy, Faculty of Allied Health Sciences, Naresuan University, Phitsanulok, Thailand; 4 Department of Physical Therapy, School of Allied Health Sciences, University of Phayao, Phayao, Thailand; 5 Department of Radiology, Faculty of Medicine, Khon Kaen University, Khon Kaen, Thailand

**Keywords:** aging, bone mineral content, hand grip strength, muscle strength, skeletal muscle

## Abstract

**Background:**

Older adults frequently experience body composition changes—decreased lean body mass (LBM) and bone mineral content (BMC), along with increased body fat mass (FM)—which affect their health and independence. However, the need for standard complex and costly imaging modalities could delay the detection of these changes and retard treatment effectiveness. Thus, this study explored the ability of practical measures, including simple muscle strength tests and demographic data, to determine the body composition of older adults.

**Methods:**

Participants (*n* = 111, with an average age of 77 years old) were cross-sectionally assessed for the outcomes of the study, including upper limb loading during a seated push-up test (ULL-SPUT), hand grip (HG) strength test and body composition.

**Results:**

The ULL-SPUT significantly correlated with body composition (*r* or *r_s_, =* 0.370–0.781; *P* < 0.05), particularly for female participants and was higher than that found for the HG strength test (*r_s_* = 0.340–0.614; *P* < 0.05). The ULL-SPUT and HG strength test, along with gender and body mass index (BMI), could accurately determine the LBM and BMC of the participants up to 82%.

**Conclusion:**

The ULL-SPUT along with gender and BMI can be used as a practical strategy to detect the LBM and BMC of older adults in various settings. Such a strategy would facilitate timely managements (i.e. standard confirmation or appropriate interventions) in various settings.

## Introduction

Older adults commonly experience body composition changes—decreased lean body mass (LBM), bone mineral content (BMC) and increased body fat mass (FM)—which affect their overall functions and health (1–4). LBM (comprising up to 65% of body weight) is important for protection against frailty and physical dysfunction (1, 3, 5). LBM reduction is associated with a decrease in skeletal muscle mass (SMM), a major component of LBM (up to 30%–40% of the body weight) (6), which is the largest reservoir of the body’s protein and a main source of amino acids for maintaining protein synthesis in other vital tissues during periods of stress, disease, undernutrition or starvation (1, 3, 7).

Diminished SMM predisposes older people to impaired glucose metabolism, frailty, sarcopenia, dependence, falls and hospitalisation (1, 3, 8). Decreased BMC (normally comprising up to 10% of the body weight) reduces bone strength and enhances the risk of osteopenia, osteoporosis and fractures (5, 9). Excessive accumulation of body FM (> 25% of body weight) impairs muscular contraction and force generation (2, 5, 10). Consequently, body composition changes could affect the physical ability, health and independence of older people, increasing the burden of care from families and societies (11, 12).

Nonetheless, current body composition assessments using complex and costly imaging modalities, such as magnetic resonance imaging (MRI) and dual-energy X-ray absorptiometry (DXA), are generally available only in large hospitals. Thus, they are commonly prescribed for individuals with obvious body composition changes that may affect the effectiveness of treatment and management (13, 14). Netz et al. (15) suggested that the slightest physical reduction could transform a person from independence into one with a disability who needs external assistance from persons or devices. Therefore, an exploration of a practical strategy enabling the early detection of older adults who face body composition changes is important for the timely initiation of appropriate and effective management in various clinical and home-based settings.

Previous studies suggest that age, gender and body mass index (BMI) are vital determinants for the body composition of individuals (2, 16–19). Some studies also applied a simple muscle strength test, namely a handgrip (HG) strength test. The test involves small muscles of the tested hand that are susceptible to decline with ageing to additionally reflect the body composition of older adults (20–22). However, the HG strength test is performed in an opened kinetic-chain manner (i.e. no need to overcome the body weight); thus, the outcomes showed low-to-moderate correlation to body composition of older adults. Recent studies have reported the ability of upper limb loading during a seated push-up test (ULL-SPUT), a closed kinetic-chain measure involving many upper limb and upper trunk muscles, to reflect body composition in clinical and older populations (23–25). However, an investigation of older adults (24) assessed the ability of the ULL-SPUT to reflect only the SMM that was measured using a bioelectrical impedance analysis in a few numbers and as a whole group of well-functioning older participants (*n* = 40).

The bioelectrical impedance analysis has been questioned for its application as a standard measure due to the possibility of errors from many factors, such as electrode placement, skin conductibility, body temperature and participant preparation (position, overnight fasting and bladder voiding) (26). Male and female older adults also experience different rates of body composition changes due to the ageing process (9). Therefore, the present study further explored the correlation between outcomes of two simple measures (i.e. the HG strength test and ULL-SPUT) and body composition (including LBM, BMC and FM), which was assessed using DXA among male and female community-dwelling older adults.

In addition, the study formulated predictive equations to offer a practical strategy for determining the body composition of the participants using the ULL-SPUT and HG strength test, along with their demographic data.

## Methods

### Study Design and Participants

Older adults aged ≥ 65 years old, both males and females, who could come to the hospital, were cross-sectionally recruited from many communities within the Khon Kaen province (24). Eligible participants had a BMI < 30 kg/m^2^ with the ability to walk (with or without an assistive device) with no more than contact guarding assistance from another person (27), and the ability to understand and follow commands for the tests used in this study. Older adults were excluded if they presented any signs and/or symptoms that might affect their ability to participate in the study, such as musculoskeletal conditions with a pain score of > 5 out of 10 on a numeric pain-rating scale; unstable medical conditions such as hypertension, dizziness and heart disease; and conditions that make DXA assessment problematic (e.g. cancer, implants and inability to attain a correct position for the measurement) (7, 25).

Eligible participants signed written informed consent forms that were approved by the Khon Kaen University Ethics Committees for Human Research, Khon Kaen, Thailand, prior to participation in the study.

### Sample Size Calculation

A sample size calculation for a correlation study based on the data from a previous study (*r* = 0.457), with 80% power and an alpha value of 0.05, indicated that the study required at least 46 participants per group of older male and female adults (28).

### Research Protocols

Eligible participants were interviewed and assessed for their demographics, including age, body weight, height, vital signs, underlying disease and walking devices (if any). Then, they were assessed for the outcomes of the study, including the HG strength test and ULL-SPUT, in a random order by an experienced and excellent reliable assessor (intraclass correlation coefficient or ICC > 0.90). Within 7 days, participants were assessed for their body composition (i.e. LBM, BMC and FM) at a hospital by an experienced radiologist. Details of the assessments are outlined below.

### Hand Grip Strength Test

The HG strength test is a valid measure that is commonly used to reflect global muscle strength and functional mobility (*r* = −0.568–0.690; *P* < 0.001) (29, 30). The participants were assessed using the Southampton HG strength test protocols while seated in a standard armchair with their feet flat on the floor, their forearms on the armrests of the chair, the wrists over the ends of the armrests in a neutral position and the thumbs facing upward. The participants held a calibrated HG strength test dynamometer (Baseline^®^ hydraulic hand dynamometer—with an outcome accuracy > 97%) and squeezed it as hard as possible for two trials with each hand. The highest value from all trials was recorded in kilograms (31, 32).

### Upper Limb Loading During a Seated Push-Up Test

**Figure 1:**
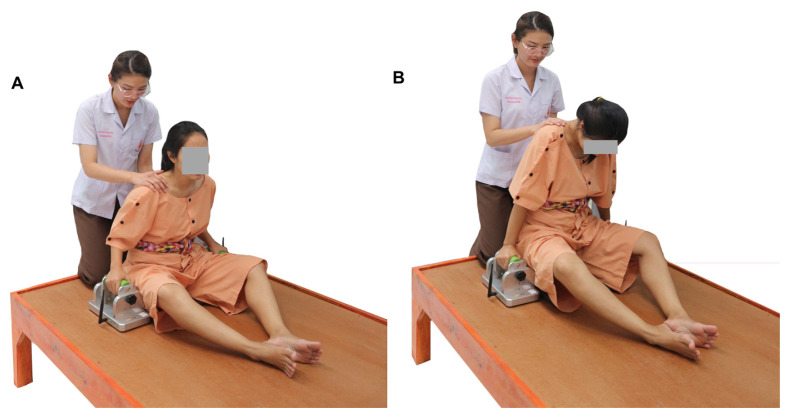
Testing protocols for the upper limb loading during a seated push-up test. A: Starting position, B: Position while lifting the body upward

The ULL-SPUT was executed using push-up loading devices that were developed from digital load cells (Model L6E3-C, 50 kg-3G with a standard calibration method based on United Kingdom Accreditation Service Lab 14: 2006, with outcome accuracy up to 0.1 kg [mini-patent application number 2103001612]) based on the concept of a digital bathroom scale as the size of clinical push-up boards (14 cm × 27 cm) (24, 25). A recent study recommended performing the ULL-SPUT in a ring-sitting position to minimise confounding factors due to muscle length and a sense of fear of falling (24). Thus, the present study assessed the ULL-SPUT while the participants were in a ring-sitting position on a hard and even surface (Figure 1A), placing both hands on the handlebars of the push-up loading devices, which were positioned slightly in front of the hips. Then, the participants pushed both hands against the devices, lifted their body up while leaning their trunk forward and bent their elbows to place their buttocks smoothly on the surface without using their lower limbs (Figure 1B). The average maximum ULL-SPUT values from the push-up loading devices over three trials were used for data analysis (24, 25).

### Body Composition Assessments

Participants were assessed for their body composition using DXA, a preferred method for research and clinical use to distinguish LBM, BMC and FM (33, 34). The outcomes showed excellent reliability (ICC = 0.997; *P* < 0.001) with excellent validity as compared with those from MRI (*r* = 0.94; *P* < 0.001). In addition, DXA is less expensive than MRI and is available in general hospitals (7, 33, 34). The participants were in a supine position on a DXA table with their arms by their sides, lower limbs extended and the toes facing upward (35). The regional and total body composition data, including LBM, BMC and FM, were automatically generated by the machine in kilograms.

### Statistical Analysis

The Kolmogorov-Smirnov and Shapiro-Wilk tests were used to assess the normality of the data distribution. Descriptive statistics were utilised to explain the demographics and findings of the study. The data of male and female participants were compared using the independent samples *t*-test for continuous data and the chi-squared test for categorical variables. The correlations were analysed using the Pearson’s correlation coefficient (*r*) for data with normal distribution and Spearman’s rank correlation coefficient (*r_s_*) for data with non-normal distribution. The levels of correlation were identified as weak (*r* or *r_s_* = 0.1–0.3), moderate (*r* or *r_s_* = 0.4–0.6), strong (*r* or *r_s_* = 0.7–0.9) and perfect (*r* or *r_s_* = 1.0) (36). The stepwise multiple linear regression analysis was utilised to formulate a predictive equation from all possible demographic and simple muscle strength measures investigated in this study (including age, gender, BMI, ULL-SPUT and HG strength test data) to determine the body composition of the participants. The results were reported as adjusted *R*^2^ and beta (*β*) coefficients. An adjusted *R*^2^ was used to identify the most appropriate equation, with an adjusted *R*^2^ of 1.0 indicating that the data perfectly fit the linear model. An adjusted *R*^2^ > 0.5 (> 50% of the variability in the outcomes) indicated an appropriate level for the predictive equation of body composition (37). A *P*-value of < 0.05 was considered statistically significant.

## Results

### Participant Characteristics

A total of 111 community-dwelling older adults (47 males and 64 females) with an average age of approximately 77 years old and a normal BMI completed the study (Table 1). Twenty-one participants (16 females [76%]) used a walking device and 62 participants (36 females [58%]) had underlying conditions ranging from one to four types (Table 1). With similar BMI, female participants had significantly lower HG strength test, ULL-SPUT, LBM and BMC, along with significantly higher FM than the male participants (*P* < 0.001). Female participants also had lower LBM and BMC, with higher FM than normal values (Table 1).

### Correlation between Simple Muscle Strength Measures (HG Strength Test and ULL-SPUT) and Body Composition of the Participants

**Figure 2:**
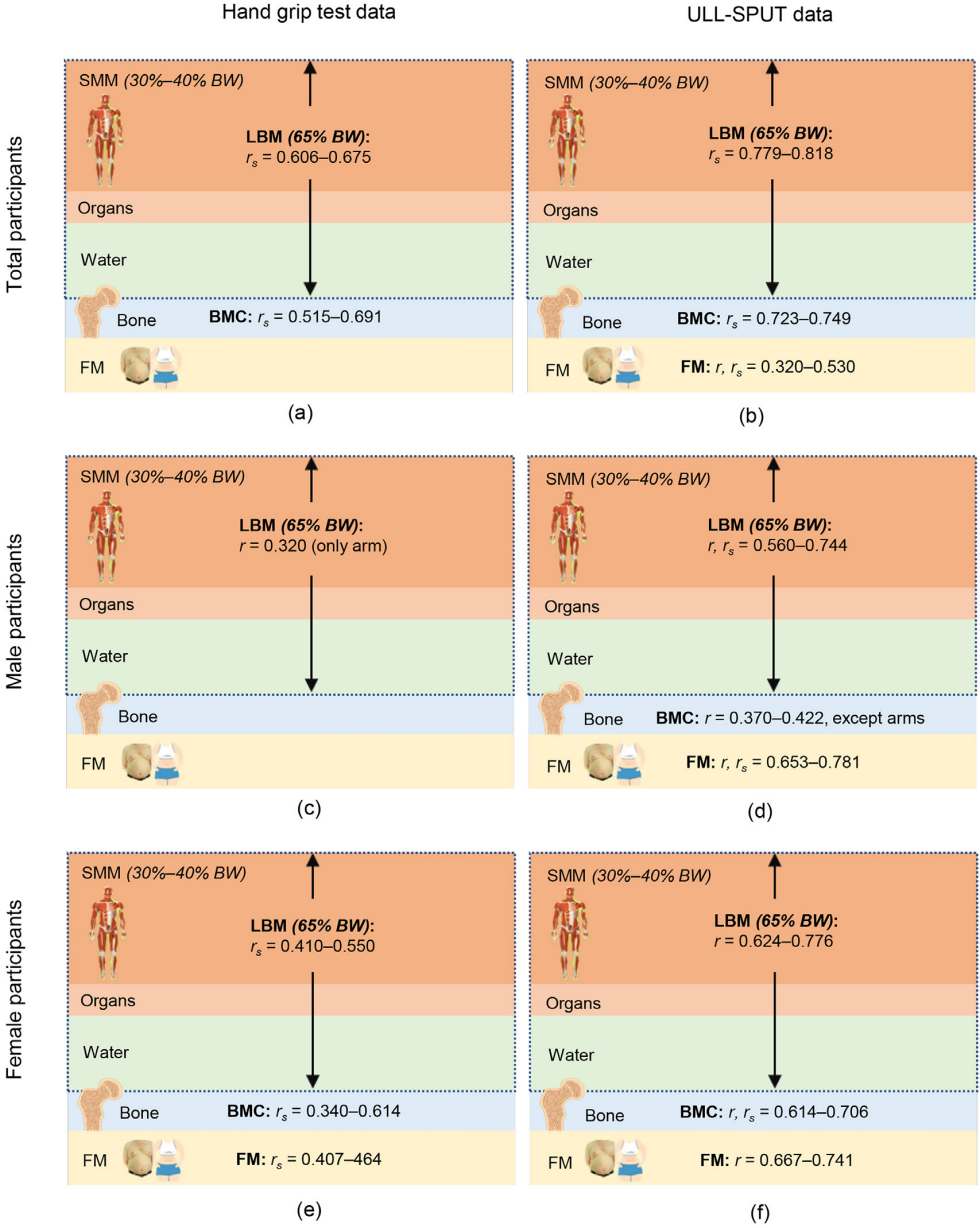
Range of correlation with handgrip data and the upper limb loading during a seated push-up test (ULL-SPUT) for the body composition Notes: LBM = lean body mass, BMC = bone mineral content, FM = fat mass, SMM = skeletal muscle mass, BW = bodyweight

In all participants, the HG strength test data showed a moderate correlation with the total and regional LBM and BMC (*r_s_* = 0.515–0.691; *P* < 0.001; Table 2, Figure 2a), whereas the ULL-SPUT data demonstrated a low-to-strong correlation with the participants’ total and regional all body composition (*r* or *r_s_* = 0.320–0.818; *P* < 0.05) (Table 2, Figure 2b). When analysed separately by gender, the ULL-SPUT data were significantly correlated with the total and regional body composition of both male and female participants (*r* or *r_s_* = 0.370–0.781; *P* < 0.05), except for FM of the upper limbs of male participants (Table 2, Figures 2d and 2f). On the contrary, the HG strength test data showed a low-to-moderate correlation with the total and regional body composition of female participants (*r_s_* = 0.340–0.614; *P* < 0.05), but for male participants, the HG strength test data correlated only with the LBM in the upper limbs (*r* = 0.320; *P* < 0.05) (Table 2, Figures 2c and 2e).

### Predictive Equations for Body Composition Using Demographic Data and Practical Measures

Of all simple demographic variables, only gender and BMI, not age, could determine the body composition of the participants (*P* < 0.05) (Table 3). Gender and BMI, in combination with the ULL-SPUT, could determine the LBM and BMC of the participants up to 82%, whereas the combination with the HG strength test could determine only the LBM of the participants (Table 3). Nevertheless, both the ULL-SPUT and HG strength test were unable to indicate the body FM of the participants (*P* > 0.05, Table 3).

## Discussion

This study explored a practical strategy, including simple demographic data and outcomes of practical measures (i.e. the ULL-SPUT and HG strength test), to determine body composition of male and female older adults. The findings indicated that the ULL-SPUT data were significantly correlated with the total and regional body composition of both male and female participants, whereas the HG strength test data were correlated only with the body composition of female participants and at a lower level than the ULL-SPUT data (Table 2). However, when taking the demographic data into account, gender and BMI in combination with the ULL-SPUT could determine the LBM and BMC, but not the body FM, of the participants (Table 3).

The high *β* coefficients from the multiple linear regression analysis confirmed the influence of gender and BMI on the body composition of the participants (Table 3). Existing evidence suggests the influence of anatomy, physiology, hormones and physical activity on the body composition of older males and females (9, 16). In females, hormonal changes after menopause result in the loss of SMM, muscle strength and BMC, with a faster rate of FM accumulation than in older male adults (9, 16, 17). Therefore, at a similar age and BMI, the female participants in the present study had significantly lower LBM and BMC, with higher FM than those of the male participants (Table 1). The body composition of female participants in the present study also showed obvious changes as compared to the normal values (LBM < 65%, BMC < 10% and FM > 25%) (5). Previous studies additionally reported that the LBM, BMC and FM occupy various proportions of the human body weight, suggesting a vital role of BMI in the determination of body composition of older adults (38, 18, 19).

The obvious body composition changes among the female participants also enabled a clear correlation between the HG strength test and the ULL-SPUT outcomes and their body composition (Table 2). In contrast, in male participants who had less change in body composition, the data showed a significant correlation with only the ULL-SPUT, a challenging measure performed in a closed kinetic-chain manner against body weight (23–25, 39). Such ability requires the SMM, a major part of LBM (Figure 2), to convert chemical energy to mechanical energy for force and power production to lift the body upward by both upper limbs and upper trunk muscles (6).

Apart from the SMM, the LBM also comprises organs and water (Figure 2), which resist muscular actions in the test. Therefore, the ULL-SPUT, along with the gender and BMI data, could determine LBM up to 82% (Table 3). With the closed association of the musculoskeletal system, the ULL-SPUT, which involves many upper limb and upper trunk muscles, also delivers a significant compressive load to the bones and across the joints, which is necessary for bone adaptation, bone mass and bone strength (40, 41). Such a challenging nature of the ULL-SPUT working against body weight also enabled outcomes of the test, along with gender, to determine the BMC, a small portion of the body composition (approximately 10% of the body weight) of the participants (Figure 2 and Table 3).

To the best of the researchers’ knowledge, only a few studies have reported the use of the ULL-SPUT as a clinical measure. These studies also found the ability of the ULL-SPUT to determine the body composition of individuals with spinal cord injuries and older people (23–25). However, data on older adults (24) were reported in a whole group of 40 participants using bioelectrical impedance analysis as a standard measure. Therefore, the present findings further confirm the ability of the ULL-SPUT to determine the body composition of older males and females.

In contrast, the HG strength test is a less demanding measure involving only the distal muscles of the tested upper limb acting in an opened kinetic-chain manner (42, 43). Therefore, the HG strength test outcomes demonstrated a clear correlation with LBM, a major part of the human body (> 65% of body weight) (5), only in female participants who exhibited an obvious change in their body composition as compared with male participants (Tables 1–3). Previous studies also reported a weak-to-moderate correlation between the HG strength test and body composition (including the SMM, the cross-sectional area of the lower limb muscles and bone mineral density) of many participants (*r* = 0.298–0.556; *P* < 0.05) (20, 22, 44).

However, both the ULL-SPUT and HG strength test data were unable to determine the body FM of the participants (Table 3). The findings reflect the characteristics of the participants. With a little evidence on the use of the ULL-SPUT in older adults, this study recruited participants with a BMI < 30 kg/m^2^, who could walk independently with or without a walking device to minimise the confounding factors and adverse events that might occur to the participants due to performing such a challenging measure. Most participants in this study were well functioning, with an average BMI of 22.54 kg/m^2^ (95% CI of 3.27) (Table 1). Such a small BMI range may limit the ability of the ULL-SPUT and HG strength test, and the data found only the gender and BMI to determine the body FM of the participants (Table 3). These assumptions are consistent with a previous report (18) that found the ability of gender, together with BMI and age, to indicate the body FM of the participants. However, this study (18) recruited participants from a wide age range (20 years old–79 years old), which enabled age to be a significant predictor of body FM.

The ability to reflect both regional and total body composition of the simple measures investigated in this study additionally suggested the effects of global physiological changes (i.e. the changes in body systems due to the ageing process occurring across the entire body systems of the participants in this study) (9). Therefore, the present findings offer a practical strategy that may enable early detection of body composition of older people who commonly experience body composition decline due to various factors, including advancing age, chronic diseases and hypoactive lifestyle (1–4). Such practical strategies may enable the distribution of effective and standard healthcare services to various clinical and home-based settings. In clinical application, the ULL-SPUT may be quantified easily using digital bathroom scales with wooden box support underneath to achieve appropriate elbow flexion angles. Then, the ULL-SPUT outcomes along with gender and BMI would identify older adults who face low LBM and BMC and need further management (i.e. standard confirmation and/or timely initiation of treatment and management) to promote the effectiveness of healthcare services for these people.

However, some limitations of this study need to be addressed. The participants in this study were Thais, whereby different races (Whites, Hispanics and Asians) have various proportions of body composition (45, 46). Other recruitment criteria, including a BMI < 30 kg/m^2^ and the ability of independent walking with or without a walking device, may limit clinical application in obese individuals with poor functional ability. The sample size was calculated based on a correlation analysis, not on a regression analysis, which may have affected the power of the study. Therefore, further studies on the use of the ULL-SPUT in older adults with a wide range of BMIs and health statuses in Asians, as well as in other races, with an appropriate number of participants covering all statistical analyses, are needed to improve the power of the findings and extend the potential benefits of the ULL-SPUT in these populations.

## Conclusion

Male and female older adults experience different rates of body composition changes that affect their health and independence. The present findings suggest the use of gender and BMI, along with the ULL-SPUT, to determine LBM and BMC of male and female community-dwelling older adults. Such a practical strategy would enable the detection of those exhibiting body composition alteration in various clinical, community, home and research settings. Then, appropriate management (e.g. standard confirmation measures and/or treatments) could be initiated in a timely manner.

## Figures and Tables

**Table 1:** Demographic data of participants and findings of the study

Variable	Total (*n* = 111)		Male (*n* = 47)		Female (*n* = 64)		*P*-value†
		
	Mean (SD)	95% CI	Mean (SD)	95% CI	Mean (SD)	95% CI	
Age (years old) a	76.24 (7.23)	74.88, 77.60	77.11 (7.29)	74.97, 79.25	75.59 (7.19)	73.80, 77.39	0.279
Body weight (kg) a	53.38 (10.15)	51.47, 55.29	59.01 (9.34)	56.27, 61.75	49.25 (8.86)	47.08, 51.42	**< 0.001** *
Height (m) a	1.53 (0.08)	1.51, 1.55	1.61 (0.06)	1.59, 1.63	1.48 (0.05)	1.47, 1.49	< 0.001*
BMI (kg/m^2^) a	22.54 (3.27)	21.93, 23.16	22.70 (3.18)	21.76, 23.63	22.43 (3.35)	21.59, 23.27	0.669
HG strength test (kg) a	18.58 (5.68)	17.51, 19.65	22.34 (4.95)	20.89, 23.79	15.81 (4.48)	14.69, 16.93	**< 0.001** *
ULL-SPUT (% body weight) a	81.79 (10.82)	79.75, 83.82	86.53 (6.09)	84.74, 88.32	78.31 (12.18)	75.27, 81.35	**< 0.001** *
Underlying diseases: yes b	62.00 (55.86)		26.00 (55.32)		36.00 (56.25)		0.992
The number of underlying diseases b, ¥							
1	27.00 (24.32)		12.00 (25.53)		15.00 (23.44)		
2	19.00 (17.12)		7.00 (14.89)		12.00 (18.75)		
3	14.00 (12.61)		5.00 (10.64)		9.00 (14.06)		
4	2.00 (1.80)		2.00 (4.26)		0.00 (0.00)		
Using a walking device^b^							
No	90.00 (81.08)		42.00 (89.36)		48.00 (75.00)		
Yes							0.056
Cane	19.00 (17.12)		5.00 (10.64)		14.00 (21.88)		
Walker	2.00 (1.80)		0.00 (0.00)		2.00 (3.13)		
LBM (% body weight)^a^							
Total	65.56 (7.31)	64.18, 66.93	69.92 (6.44)	68.03, 71.81	62.56 (6.20)	60.81, 63.91	**< 0.001** *
Arms	7.28 (1.14)	7.06, 7.49	8.15 (1.03)	7.85, 8.45	6.64 (0.73)	6.46, 6.82	**< 0.001** *
Trunk	31.89 (4.02)	31.14, 32.65	33.73 (3.69)	32.65, 34.82	30.54 (3.73)	29.61, 31.47	**< 0.001** *
Legs	21.05 (2.36)	20.60, 21.49	22.56 (1.94)	21.99, 23.14	19.94 (2.00)	19.44, 20.44	**< 0.001** *
BMC (% body weight)^a^							
Total	3.43 (0.70)	3.30, 3.56	4.01 (0.65)	3.82, 4.21	3.00 (0.34)	2.92, 3.08	**< 0.001** *
Arms	0.47 (0.12)	0.45, 0.50	0.58 (0.10)	0.55, 0.61	0.39 (0.05)	0.38, 0.41	< 0.001*
Trunk	0.92 (0.20)	0.88, 0.96	1.06 (0.21)	1.00, 1.12	0.82 (0.11)	0.79, 0.85	**< 0.001** *
Legs	1.24 (0.30)	1.18, 1.29	1.50 (0.25)	1.43, 1.58	1.04 (0.15)	1.01, 1.08	**< 0.001** *
FM (% body weight)^a^							
Total	30.76 (7.81)	29.32, 32.26	25.93 (6.97)	23.88, 27.98	34.36 (6.36)	32.77, 35.95	**< 0.001** *
Arms	3.64 (1.09)	3.43, 3.85	2.14 (0.62)	2.56, 2.92	4.30 (0.88)	4.08, 4.52	**< 0.001** *
Trunk	15.56 (5.02)	14.62, 16.51	13.33 (4.83)	11.91, 14.74	17.20 (4.53)	16.07, 18.34	**< 0.001** *
Legs	10.11 (2.65)	9.61, 10.61	8.38 (2.03)	7.78, 8.98	11.38 (2.35)	10.80, 11.96	**< 0.001** *

Notes:

a The data between males and females;

† *P*-value were compared using the independent-samples *t*-tests;

b *n* (percent) and compared using the chi-squared test;

¥ Health problems of participants included diabetes, hypertension, hyperlipidemia, chronic kidney disease, osteoarthritis, anemia, heart disease, gout, hyperthyroid and asthma;

* significant value; 95% CI **=** 95% confidence interval; ULL-SPUT = upper limb loading during a seated push-up test; BMI = body mass index; HG = hand grip; LBM = lean body mass; BMC= bone mineral content; FM= fat mass

**Table 2:** Correlation between body composition, and data of the hand-grip test (HG) and the upper limb loading during a seated push-up test (ULL-SPUT)

Variables (kg)	Total (*n* = 111)		Male (*n* = 47)		Female (*n* = 64)	
		
	HG (kg) *r* (95% CI)	ULL-SPUT (kg) *r* (95% CI)	HG (kg) *r* (95% CI)	ULL-SPUT (kg) *r* (95% CI)	HG (kg) *r* (95% CI)	ULL-SPUT (kg) *r* (95% CI)
LBM						
Total	**0.653**β **(0.53, 0.75)**	**0.818**β **(0.74, 0.87)**	0.202α (−0.09, 0.46)	**0.725**α **(0.55, 0.84)**	**0.513**β **(0.30, 0.68)**	**0.716**α (0.57, 0.82)
*P*-value	**< 0.001**	**< 0.001**	0.174	**< 0.001**	**< 0.001**	**< 0.001**
Upper limbs	0.675β (0.56, 0.77)	0.814β (0.74, 0.87)	0.320α (0.04, 0.56)	0.560α (0.33, 0.73)	0.506β (0.29, 0.67)	0.776α (0.66, 0.86)
*P*-value	< 0.001	< 0.001	0.028	< 0.001	< 0.001	< 0.001
Lower limbs	**0.643**β **(0.52, 0.74)**	**0.816**β **(0.74, 0.87)**	0.172α (−0.12, 0.44)	**0.744**α **(0.58, 0.85)**	**0.550**β **(0.35, 0.71)**	0.672α **(0.51, 0.79)**
*P*-value	**< 0.001**	**< 0.001**	0.249	**< 0.001**	**< 0.001**	**< 0.001**
Trunk	0.606β **(0.47, 0.71)**	**0.779**β **(0.69, 0.84)**	0.095β (−0.21, 0.38)	**0.608**β **(0.38, 0.77)**	**0.410**β **(0.18, 0.60)**	**0.624**α **(0.45, 0.75)**
*P*-value	**< 0.001**	**< 0.001**	0.526	**< 0.001**	**0.001**	< 0.001
BMC						
Total	**0.615**β **(0.48, 0.72)**	**0.749**β **(0.65, 0.82)**	−0.009α (−0.30, 0.28)	**0.400**α **(0.13, 0.62)**	**0.437**β **(0.21, 0.62)**	**0.677**β **(0.51, 0.79)**
*P*-value	**< 0.001**	**< 0.001**	0.950	**0.005**	**< 0.001**	**< 0.001**
Upper limbs	**0.691**β **(0.58, 0.78)**	**0.731**β **(0.63, 0.81)**	0.136α (−0.16, 0.41)	0.283α (−0.01, 0.53)	**0.614**β (0.43, 0.75)	**0.706**α (0.6, 0.81)
*P*-value	**< 0.001**	**< 0.001**	0.363	0.054	**< 0.001**	**< 0.001**
Lower limbs	**0.672**β^**^ **(0.55, 0.77)**	**0.723**β^**^ **(0.62, 0.80)**	0.146α (−0.15, 0.41)	**0.370**α **(0.09, 0.59)**	**0.549**β **(0.34, 0.70)**	**0.637**α **(0.46, 0.74)**
*P*-value	**< 0.001**	**< 0.001**	0.334	**0.010**	**< 0.001**	**< 0.001**
Trunk	**0.515**β **(0.36, 0.64)**	**0.741**β **(0.64, 0.82)**	−0.164α (−0.43, 0.13)	**0.422**α **(0.15, 0.63)**	**0.340**β **(0.10, 0.55)**	**0.614**α **(0.43, 0.75)**
*P*-value	**< 0.001**	**< 0.001**	0.269	**0.003**	**0.006**	**< 0.001**
FM						
Total	0.034β (−0.1, 0.22)	**0.522**α **(0.37, 0.65)**	−0.237α (−0.49, 0.05)	**0.732**α **(0.56, 0.84)**	**0.464**β **(0.24, 0.64)**	**0.741**α **(0.61, 0.84)**
*P*-value	0.727	**< 0.001**	0.109	**< 0.001**	**< 0.001**	**< 0.001**
Upper limbs	−0.053β (−0.24, 0.14)	**0.320**α **(0.14, 0.48)**	−0.159α (−0.43, 0.14)	**0.653**α **(0.45, 0.79)**	**0.433**β **(0.20, 0.62)**	**0.693**α (0.54, 0.80)
*P*-value	0.583	**0.001**	0.287	**< 0.001**	**0.001**	**< 0.001**
Lower limbs	−0.008β (−0.20, 0.18)	**0.480**β **(0.32, 0.62)**	−0.230β (−0.49, 0.07)	**0.781**β **(0.63, 0.88)**	**0.407**β **(0.17, 0.60)**	**0.667**α **(0.50, 0.79)**
*P*-value	0.973	**< 0.001**	0.119	**< 0.001**	**< 0.001**	**< 0.001**
Trunk	0.042β (−0.15, 0.23)	**0.530**α **(0.38, 0.65)**	−0.260α (−0.51, −0.03)	**0.724**α **(0.55, 0.84)**	**0.419**β **(0.19, 0.61)**	**0.696**α **(0.54, 0.80)**
*P*-value	0.662	**< 0.001**	0.077	**< 0.001**	**0.001**	**< 0.001**

Notes: *n* = number; 95% CI = 95% confidence interval; kg = kilogram;

α analysed using the Pearson’s correlation coefficients;

β analysed using the Spearman’s rank correlation coefficients;

LBM = lean body mass; BMC = bone mineral content; FM = fat mass

**Table 3:** Multiple linear regression to determine body composition using demographic data and practical measures investigated in this study, including the HG strength test and upper limb loading during a seated push-up test (ULL-SPUT)

Predictive variable	Beta weights	95% CI	*P-*value	Predictive formulae	Adjusted *R*^2^
**Total LBM (g)**					
For the HG strength test					
Gender	−8,769.96	10,094.90, −7,445.01	< 0.001	−8,769.96 (gender) + 776.47 (BMI) + 227.56 (HG) + 26,874.06	0.812
BMI (kg/m^2^)	776.47	609.34, 943.59	< 0.001		
HG data (kg)	227.56	110.54, 344.59	< 0.001		
For the ULL-SPUT data					
Gender	−7,666.40	−9,170.22, −6,162.58	< 0.001	−7,666.40 (gender) + 371.07 (BMI) + 222.50 (ULL-SPUT) + 28,740.11	0.823
BMI (kg/m^2^)	371.07	122.72, 619.42	0.004		
ULL-SPUT (kg)	222.50	129.85, 315.16	< 0.001		
**Total BMC (g)**					
For the HG strength test					
Gender	−852.35	−953.34, −751.35	< 0.001	− 852.35 (gender) + 43.73 (BMI) + 2,194.23	0.743
BMI (kg/m^2^)	43.73	28.40, 59.06	< 0.001		
HG data (kg)	9.59	−1.18, 20.35	0.80		
For the ULL-SPUT data					
Gender	− 637.40	−754.03, −520.77	< 0.001	−637.40 (gender) + 18.70 (ULL-SPUT) + 2020.95	0.767
BMI (kg/m^2^)	13.01	−9.62, 35.65	0.257		
ULL-SPUT (kg)	18.70	13.25, 24.14	< 0.001		
**Total FM (g)**					
For the HG strength test					
Gender	1,936.21	942.38, 2,930.04	< 0.001	1,936.21 (gender) + 1,593.00 (BMI) − 22,309.80	0.802
BMI (kg/m^2^)	1,593.00	1,442.14, 1,743.86	< 0.001		
HG data (kg)	−30.92	−138.21, 76.38	0.569		
For the ULL-SPUT data					
Gender	1,936.21	942.38, 2,930.04	< 0.001	1,936.21 (gender) + 1,593.00 (BMI) − 22,309.80	0.802
BMI (kg/m^2^)	1,593.00	1,442.14, 1,743.86	< 0.001		
ULL-SPUT (kg)	67.36	−19.45, 154.16	0.127		

Note: gender was indicated as 1 for male and 2 for female; 95% CI = 95% confidence interval; HG = handgrip; ULL-SPUT = upper limb loading during a seated push-up test; LBM = lean body mass; BMC = bone mineral content; BMI = body mass index; FM= fat mass
